# Comparison of lidocaine alone or in combination with a local nerve block of ethanol, bupivacaine liposome suspension, or oral meloxicam to extend analgesia after scoop dehorning in Holstein calves

**DOI:** 10.3168/jdsc.2021-0178

**Published:** 2022-03-03

**Authors:** Miriam S. Martin, Michael D. Kleinhenz, Abbie V. Viscardi, Shawnee R. Montgomery, Charley A. Cull, Jon E. Seagren, Kelly F. Lechtenberg, Johann F. Coetzee

**Affiliations:** 1 Department of Anatomy and Physiology, Kansas State University College of Veterinary Medicine, Manhattan 66506; 2 Department of Clinical Sciences, Kansas State University College of Veterinary Medicine, Manhattan 66506; 3 Midwest Veterinary Services Inc., Oakland, NE 68045

## Abstract

The American Veterinary Medical Association recommends the use of practices that reduce or eliminate pain and discomfort associated with dehorning. Identification of an effective, long-acting local anesthetic that is practical for producers to implement and reduces pain from dehorning would benefit animal welfare. Thirty-two Holstein bulls and heifers were enrolled. The objective of this study was to compare the efficacy and duration of activity of bupivacaine liposome suspension (BUP; n = 8), ethanol (ETH; n = 8), or meloxicam (LID + MEL; n = 8) co-administered with lidocaine compared with lidocaine only (LID; n = 8), and to quantify their effect on pain biomarkers and behaviors after scoop dehorning with cauterization in approximately 20-wk-old calves. Outcome variables collected included infrared thermography (IRT), mechanical nociceptive threshold (MNT), visual analog scale (VAS) scoring, and blood sampling for serum cortisol and prostaglandin E_2_ metabolites (PGEM). There was evidence of a sex effect for MNT, with bulls demonstrating a higher threshold (13.74 kgf) compared with heifers (12.12 kgf). There was a treatment by time interaction for cortisol concentrations (ng/mL). At 2 h, the BUP group had higher cortisol values (17.32 ng/mL) than the LID + MEL group (3.10 ng/mL). Heifers also had higher mean cortisol values (13.88 ng/mL) compared with bulls (6.96 ng/mL). There was a treatment by time interaction for PGEM concentration. Calves in the LID + MEL group had lower PGEM values at 4 and 8 h (10.23 and 9.12 pg/mL) than at −24, 0, and 0.5 h (20.38, 27.27, and 22.59 pg/mL, respectively). At 4 h, the LID + MEL group had lower PGEM concentrations (10.23 pg/mL) than the ETH group (27.08 pg/mL). At 8 h, the LID + MEL group had lower PGEM concentrations (9.12 pg/mL) than both the ETH and BUP groups (24.80 and 20.52 pg/mL). Thus, LID + MEL reduced cortisol and prostaglandin metabolite concentrations more effectively than ETH + LID or BUP + LID administered as a local infiltration and cornual block, respectively, before scoop dehorning followed by cauterization. The treatments administered in the present study did not seem to extend the duration of analgesia beyond the currently recommended multimodal approach, including local anesthesia and systemic analgesia such as lidocaine and meloxicam. Evidence from the current study suggests that sex influences pain biomarkers such as nociceptive threshold and cortisol concentration, with males having a higher nociceptive threshold and lower cortisol responses.

## Highlights

• Extending the duration of analgesia after dehorning would benefit animal welfare.
• Lidocaine + meloxicam reduced cortisol and prostaglandin E2 most effectively.
• Sex of calf influenced pain biomarkers such as nociceptive threshold and cortisol.
• Male calves had a higher nociceptive threshold and lower cortisol response than female calves.

## Graphical Abstract

**Figure:**
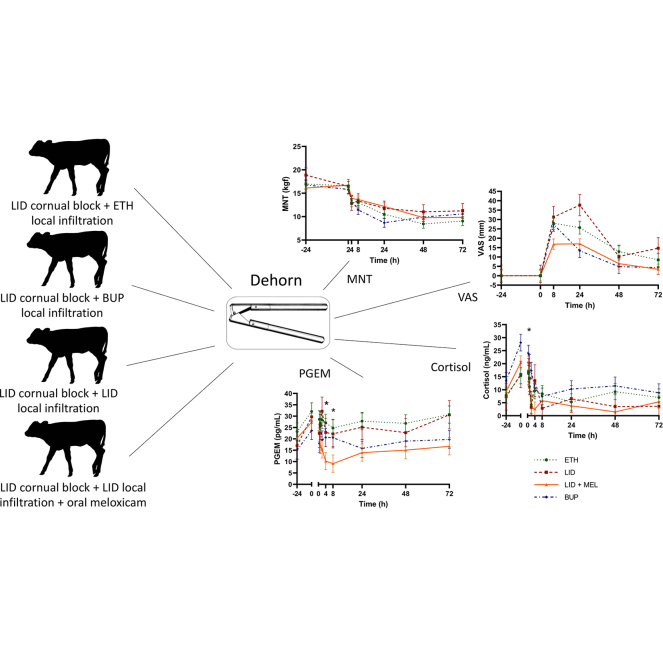
**Summary:** The AVMA recommends the use of procedures that reduce ain and discomfort with dehorning. We compared the efficacy and duration of activity of bupivacaine liposome suspension (BUP), ethanol (ETH), or meloxicam (MEL) co-administered with lidocaine (LID) compared with LID only, measured via pain biomarkers and behaviors after scoop dehorning with cauterization in approximately 20-wk-old calves. Outcomes included infrared thermography, mechanical nociceptive threshold (MNT), visual analog scale (VAS) scoring, and blood sampling for serum cortisol and prostaglandin E2 metabolites (PGEM). The LID + MEL treatment reduced cortisol and PGEM more effectively than ETH + LID or BUP + LID administered as a local infiltration and cornual block, respectively, before dehorning.

The American Veterinary Medical Association (**AVMA**) recognizes that dehorning of cattle increases safety during handling and transportation, allows cattle to take up less bunk space, and reduces carcass bruising (AVMA, 2014). Because dehorning causes pain and discomfort, the AVMA recommends the use of procedures and practices that reduce or eliminate these effects (AVMA, 2019). The American Association of Bovine Practitioners (AABP) recommends that dehorning be performed at the youngest age possible (AABP, 2019), and the National Dairy FARM Program Animal Care Reference Manual (version 4) requires that calves be disbudded by 8 wk of age (National Milk Producers Federation, 2020). However, male calves with dairy influence destined for beef production may not be raised according to these standards and may be dehorned at a later age. Many sources recommend the use of a local anesthetic before dehorning to reduce behavioral and physiological pain responses (Stafford and Mellor, 2005). Additionally, the administration of nonsteroidal anti-inflammatory drugs (**NSAIDs**) has been shown to provide extended postoperative analgesia following dehorning (Allen et al., 2013; Kleinhenz et al., 2017). Anesthesia of the horn bud for dehorning is achieved by injecting local anesthetic over branches of the cornual nerve. With experienced staff, this method is 88 to 100% effective (Winder et al., 2018). An alternative method to the cornual block outlined in Bates et al. (2019) is local site infiltration of an anesthetic rostromedial and caudomedial to the horn bud, which has been found to result in a lesser behavioral response during dehorning compared with a cornual block. Lidocaine is the most widely used local anesthetic but has a limited duration of activity (Riviere and Papich, 2018). Ethanol has been shown to result in less pressure sensitivity than lidocaine after dehorning (Tapper, 2011). Bupivacaine has a longer duration of action but a slower onset than lidocaine (Riviere and Papich, 2018; Martin et al., 2022). Administering lidocaine and bupivacaine has been found to result in a rapid onset and prolonged duration of action (Best et al., 2015). Finding the most effective local anesthetic that is practical for producers to implement and reduces pain from dehorning would be beneficial to animal welfare. The null hypothesis of this study was no difference in efficacy, onset, and duration of strategies to extend analgesia following scoop dehorning followed by cauterization. The study objectives were to determine the efficacy, onset, and duration of strategies to extend analgesia for bupivacaine + lidocaine, ethanol + lidocaine, or lidocaine + meloxicam compared with lidocaine only, and to determine which local anesthetic or combination of local anesthetic + NSAID most effectively reduces pain biomarkers and pain behaviors following scoop dehorning.

The Midwest Veterinary Services Institutional Animal Care and Use Committee reviewed and approved the experimental protocol for this project (IACUC# MCL 20055). The study took place in August 2020 at the Midwest Veterinary Services research facility near Oakland, Nebraska. Calves were group housed in outdoor pens of size exceeding the guidelines for calf housing in the *Guide for Care and Use of Agricultural Animals in Research and Teaching* (FASS, 2020). Calves were fed a grain diet formulated to meet or exceed the nutritional requirements set by the National Research Council (NRC, 2016). A total of 32 male and female Holstein calves (9 bulls and 23 heifers) weighing 233 ± 11 kg, approximately 20 wk of age, all weaned, vaccinated, horned, and intact, were randomized using the RAND function in Excel (2016, Microsoft Corp.) by horn bud width and then enrolled into 1 of 4 experimental treatment groups: (1) lidocaine (lidocaine HCl 2% injection; MWI) cornual block + ethanol (ethanol 200 proof, Decon Laboratories) local infiltration + oral placebo (**ETH**; n = 8; 4 heifers and 4 bulls); (2) lidocaine cornual block + lidocaine local infiltration + oral placebo (**LID**; n = 8; 6 heifers and 2 bulls); (3) lidocaine cornual block + lidocaine local infiltration + oral meloxicam (1 mg/kg; Zydus Pharmaceuticals; **LID + MEL**; n = 8; 6 heifers and 2 bulls); (4) lidocaine cornual block + bupivacaine liposome suspension (Nocita, Elanco) local infiltration + oral placebo (**BUP**; n = 8; 7 heifers and 1 bull).

Calves were administered their treatment 10 min before the dehorning procedure. The local anesthetic block for dehorning consisted of a cornual nerve block and a local block of the horn buds. For the cornual nerve block, 4 to 5 mL of local anesthetic was injected halfway between the lateral canthus of the eye and horn just ventral to the frontal crest on each side of the head. For the local block of the horn buds, as described in Bates et al. (2019), 1 mL of local anesthetic was injected laterally and caudally to the horn bud (12–14 mL total volume injected). The oral meloxicam tablets (Zydus Pharmaceuticals Inc.) were placed in a gelatin capsule (Torpac Inc.) and administered via a bolus gun at a dosage of 1 mg/kg. The oral placebo was lactose monohydrate powder (ThermoFisher Scientific), which is the binder used in meloxicam tablets; it was also placed in a gelatin capsule (Torpac Inc.) and administered via a bolus gun. At 5 min before dehorning, the local anesthetic block was tested by pricking the skin immediately adjacent to the horn with a hypodermic needle. If the animal responded to the needle stick (i.e., was not anesthetized), the local anesthetic block would have been repeated, although no calves on study responded in this way. Calves were dehorned using a Barnes dehorning instrument (Stone Manufacturing & Supply Co.). Following scoop dehorning, calves were cauterized using a preheated electric dehorning iron (Stone Manufacturing & Supply Co.) placed on the horn tissue for approximately 15 to 20 s per horn bud. All dehorning procedures were performed by a single experienced veterinarian (CC).

Outcome variables were collected at −24, 0, 0.5, 1, 2, 4, 8, 24, 48, and 72 h post-dehorning, with mechanical nociceptive testing beginning at 2 h and visual analog scale scoring beginning at 8 h following dehorning. Outcome variables collected included infrared thermography (**IRT**), mechanical nociceptive threshold (**MNT**), visual analog scale (**VAS**) scoring, and blood sampling for serum cortisol and prostaglandin E_2_ metabolites (**PGEM**). All trained evaluators were masked to treatment for the duration of the study.

Blood samples for serum cortisol and PGEM determination were collected from the jugular vein via venipuncture. The whole-blood samples were immediately transferred to tubes (Vacutainer, BD Diagnostics) containing either no additive for cortisol or EDTA anticoagulant for PGEM determination. At the end of each collection time point, approximately 30 min after collection began, blood samples were centrifuged for 10 min at 3,000 × *g*; then, serum and plasma were collected, placed in cryovials via transfer pipette, and stored at −80°C.

The IRT images captured the medial canthus of the left eye using a research-grade infrared camera (Fluke TiX580, Fluke Corp.) using calibration and methods described in Kleinhenz et al. (2017). Infrared images were analyzed using research-specific computer software (SmartView v. 4.3, Fluke Thermography) to determine maximum and minimum temperatures.

A handheld pressure algometer (Wagner Instruments) was used for MNT determination using methods and locations described in Kleinhenz et al. (2017). A force was applied perpendicularly at a rate of approximately 1 kg of force (**kgf**) per second at 5 locations, consisting of 2 points (1 laterally and 1 caudally) around each horn at the hair–horn junction and a point in the center of the forehead. A withdrawal response was indicated by an overt movement away from the applied pressure algometer. The calves were blindfolded to prevent any sudden movements, and MNT values were recorded by a second investigator to prevent bias by the investigator performing the MNT collection.

A VAS score was assigned by an evaluator masked to treatment allocations using the scoring methods described in detail in Martin et al. (2020). The VAS used was a 100-mm (10-cm) line anchored at each end by descriptors of “no pain” or “severe pain.” The evaluator marked the line between the 2 descriptors to indicate the pain intensity. A millimeter scale was used to measure the score from the zero anchor point to the evaluator's mark.

Serum cortisol concentrations were determined using a commercially available cortisol coated tube RIA kit (cat. no. 07-221106-R; MP Biomedicals) following manufacturer specifications with minor modifications as described by Martin et al. (2022). The standard curve was extended to include 1 and 3 ng/mL by diluting the 10 and 30 ng/mL manufacturer-supplied standards 1:10, respectively. The standard curve ranged from 1 to 300 ng/mL. Low (25 ng/mL) and high (150 ng/mL) quality control samples were run at the beginning and end of each set to determine interassay variability. Tubes were counted on a gamma counter (Wizard2, PerkinElmer) for 1 min. The raw data file was then uploaded onto MyAssays Desktop software (version 7.0.211.1238) for concentration determination. Standard curves were plotted as a 4-parameter logistic curve. Samples with a coefficient of variation (**CV**) >18% were reanalyzed. The project average for serum cortisol intra- and interassay CV were 14.50% and 14.87%, respectively.

Prostaglandin E_2_ metabolites were analyzed using a commercially available ELISA kit (cat. no. 514531; Cayman Chemical) following manufacturer specifications with minor modifications, as described in Martin et al. (2022). Samples were diluted 1:2 and run in duplicate. Absorbance was measured at 405 nm after 60 min of development (SpectraMax i3; Molecular Devices). The standard curve ranged from 0.39 to 50 pg/mL. Sample results were excluded if the raw read exceeded the raw read of the highest standard (standard 1; 50 pg/mL) or was below the lowest acceptable standard. Any individual sample outside the standard curve or a CV >15% were reanalyzed. The project-average PGEM intra- and interassay CV were 16.40% and 13.55%, respectively.

Mechanical nociceptive threshold was used to determine the number of calves needed per treatment group, as previously described (Heinrich et al., 2010). The study was designed to have power exceeding 0.80, assuming a difference in effect size (Δ) of 0.51, a standard error (σ) of 0.13, and a statistical inference level (α) of 0.05. Based on this calculation, a sample size of 8 animals per treatment group was determined. Concentrations of serum cortisol and PGEM were log-transformed for normality before statistical analysis. Responses (i.e., IRT, MNT, VAS, serum cortisol, and PGEM) were analyzed using linear regression with repeated measures, with calf as the experimental unit. Calves nested in a treatment group were designated as a random effect, with treatment, time, treatment × time interaction, and sex designated as fixed effects. *F*-Tests were used to test the significance of main effects and interactions. If significant overall differences were identified, pairwise comparisons were performed using the Tukey honestly significant difference (HSD) test. Statistics were performed using statistical software (JMP Pro 15.1.0; SAS Institute Inc.). Statistical significance was set a priori at *P* ≤ 0.05. Data are presented as least squares means.

Treatment means for all the outcome variables collected are outlined in Table 1. No evidence was found of a treatment effect (*P* = 0.25), treatment × time interaction (*P* = 0.25), or sex effect (*P* = 0.15) for IRT. However, there was a time effect (*P* < 0.01), with 0-h and 24-h IRT measurements (33.57 and 33.71°C; 95% CI: 33.20 to 34.08°C) being less than readings at −24, 2, 4, 8, and 72 h (>34.73°C; 95% CI: 34.25 to 37.79°C; *P* < 0.01).

**Table 1:** Least squares means (95% CI) of outcome variables by treatment

Variable^1^	Treatment (Trt)^2^				*P*-value			
	ETH	LID	LID + MEL	BUP	Trt	Time	Trt ×Time	Sex
Mean IRT (°C)	34.92	34.96	35.15	34.82	0.25	<0.01	0.25	0.15
95% CI	34.65 to 35.20	34.51 to 35.41	34.92 to 35.38	34.52 to 35.12				
Mean MNT (kgf)	12.51	13.66	13.17	12.39	0.63	<0.01	0.11	0.04
95% CI	11.18 to 13.84	11.47 to 15.85	12.04 to 14.29	10.93 to 13.84				
Change in MNT (%)	−25.22	−26.27	−13.30	−23.64	0.31	<0.01	0.09	0.16
95% CI	−37.09 to −13.34	−45.81 to −6.74	−23.34 to −3.26	−36.61 to −10.67				
Mean VAS (1–100 mm)	12.52	15.55	7.11	8.08	0.08	<0.01	0.09	0.41
95% CI	8.29 to 16.75	8.60 to 22.50	3.53 to 10.69	3.47 to 12.69				
Mean cortisol (ng/mL)	9.45	8.77	8.31	15.14	0.06	<0.01	0.03	<0.01
95% CI	5.97 to 12.93	2.99 to 14.55	5.36 to 11.27	11.31 to 18.98				
Mean PGEM (pg/mL)	27.65	25.08	16.81	19.88	<0.01	<0.01	<0.01	0.27
95% CI	22.57 to 32.72	16.74 to 33.43	12.52 to 21.10	14.33 to 25.42				

1 IRT = infrared thermography; MNT = mechanical nociceptive threshold; kgf = kilograms of force; VAS = visual analog scale; and PGEM = prostaglandin E_2_ metabolite concentration.

2 ETH = lidocaine cornual block + ethanol local infiltration + oral placebo; LID = lidocaine cornual block + lidocaine local infiltration + oral placebo; LID + MEL = lidocaine cornual block + lidocaine local infiltration + oral meloxicam; BUP = lidocaine cornual block + bupivacaine liposome suspension local infiltration + oral placebo.

No evidence was found of a treatment effect (*P* = 0.63) or a treatment × time interaction (*P* = 0.11) for mean MNT. However, there was a time effect (*P* < 0.01), with thresholds at 24, 48, and 72 h (10.80, 9.78, and 10.18 kgf; 95% CI: 8.68 to 11.91 kgf) being lower those at than at 4 and 8 h (13.31 and 12.87 kgf; 95% CI: 11.76 to 14.42 kgf), which were all lower than those at −24 and 2 h (17.18 and 16.39 kgf; 95% CI: 15.28 to 18.29 kgf; *P* < 0.01). There was evidence of a sex effect (*P* = 0.04; Figure 1), with bulls having a higher threshold (13.74 kgf; 95% CI: 12.41 to 15.10 kgf) than heifers (12.12 kgf; 95% CI: 11.23 to 13.01 kgf).

**Figure 1:**
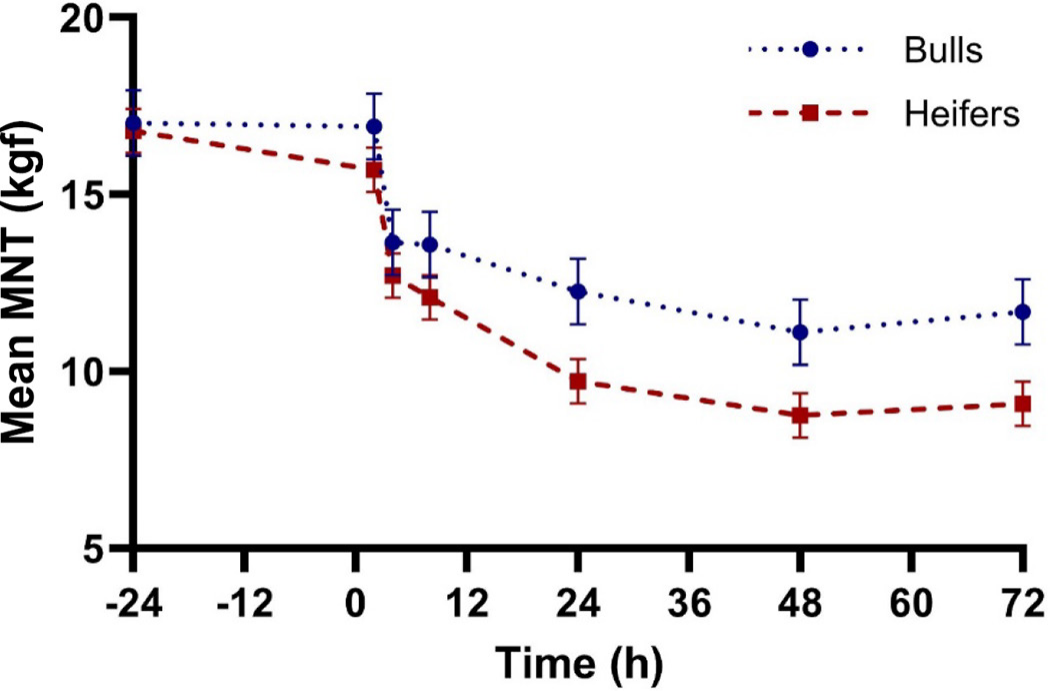
Mean mechanical nociceptive threshold (MNT) values measured in kilograms of force (kgf) over the study duration by sex of calf. Error bars indicate SEM.

There was no evidence of a significant treatment × time interaction (*P* = 0.08) for percent change from baseline MNT. For ETH, thresholds decreased at 24, 48, and 72 h (−36.72, −49.13, and −45.71%, respectively; 95% CI: −60.07 to −22.36%) relative to −24 and 2 h (0 and −0.85%; 95% CI: −15.21 to 14.36%; *P* < 0.01). For LID, thresholds decreased at 48 and 72 h (−40.23 and −39.41%; 95% CI: −63.79 to −15.85%) relative to −24 h (1.61%; 95% CI: −21.94 to 25.17%; *P* < 0.04). For LID + MEL, thresholds decreased at 24, 48, and 72 h (−19.63, −35.27, and −34.45%, respectively; 95% CI: −46.31 to −7.77%) relative to −24 and 2 h (2.61 and 10.70%; 95% CI: −9.25 to 22.54%; *P* < 0.01). For BUP, thresholds decreased at 8, 24, 48, and 72 h (−29.87, −47.23, −38.89, and −34.64%, respectively; 95% CI: −62.49 to −14.61%) relative to −24 h (3.64%; 95% CI: −11.62 to 18.90%; *P* < 0.01). There was no evidence of a sex effect for percent change from baseline MNT (*P* = 0.16).

There was no evidence of a significant treatment × time interaction (*P* = 0.09) for VAS. For ETH, VAS increased at 8 and 24 h (28 and 25.75 mm; 95% CI: 18.89 to 34.85 mm) relative to −24, 0 and 72 h (0, 0, and 8.5 mm, respectively; 95% CI: 0 to 15.35 mm; *P* < 0.01). For LID, VAS increased at 8 and 24 h (31.33 and 37.67 mm; 95% CI: 20.10 to 48.89 mm) relative to −24 and 0 h (0 and 0 mm; 95% CI: 0 to 10.89 mm; *P* < 0.03). For LID + MEL, VAS increased at 8 and 24 h (16.84 and 16.99; 95% CI: 11.31 to 22.53 mm) relative to −24, 0, and 72 h (0, 0, and 3.43 mm; 95% CI: 0 to 9.14 mm; *P* < 0.04). For BUP, VAS increased at 8 h (27.37 mm; 95% CI: 20.28 to 34.46 mm) relative to −24, 0, 48, and 72 h (0, 0, and 4.37 mm; 95% CI: 0 to 11.46 mm; *P* < 0.01). There was no evidence of a sex effect for VAS (*P* = 0.41).

We did identify a treatment × time interaction for cortisol (*P* = 0.03; Figure 2). For LID + MEL, cortisol was higher at 0 and 0.5 h (20.48 and 20.39 ng/mL; 95% CI: 15.44 to 25.43 ng/mL) relative to 2, 4, 24, and 48 h (3.10, 2.60, 5.80, and 3.82 ng/mL, respectively; 95% CI: 0 to 10.93 ng/mL; *P* < 0.02). At 2 h, BUP had higher cortisol (17.32 ng/mL; 95% CI: 10.98 to 23.67 ng/mL) relative to LID + MEL (3.10 ng/mL; 95% CI: 0 to 8.05 ng/mL; *P* = 0.03). There was also a sex effect for cortisol (*P* < 0.01). Heifers had higher mean cortisol (13.88 ng/mL; 95% CI: 11.54 to 16.22 ng/mL) than bulls (6.96 ng/mL; 95% CI: 3.41 to 10.50 ng/mL).

**Figure 2:**
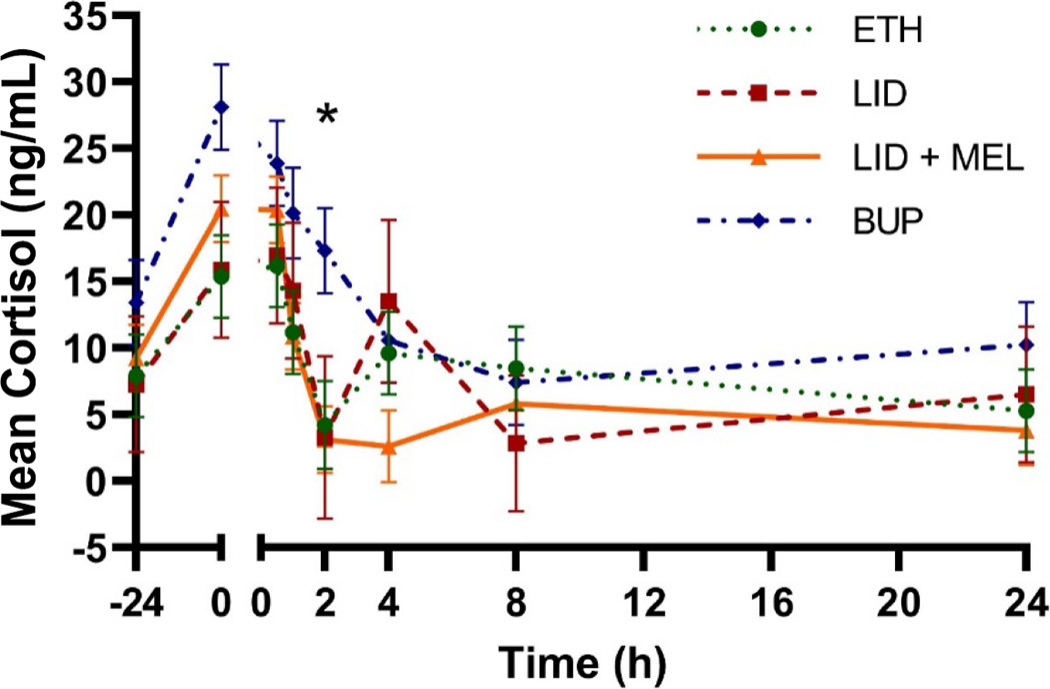
Mean cortisol concentrations (ng/mL) over the first 24 h of the study for each of the 4 treatment groups. No significant differences were observed beyond 24 h. Treatment: ETH = lidocaine cornual block + ethanol local infiltration + oral placebo; LID = lidocaine cornual block + lidocaine local infiltration + oral placebo; LID + MEL = lidocaine cornual block + lidocaine local infiltration + oral meloxicam; BUP = lidocaine cornual block + bupivacaine liposome suspension local infiltration + oral placebo. Error bars indicate SEM. *Denotes time points where a significant difference (*P* ≤ 0.05) was observed between at least 2 treatment groups.

There was a treatment × time interaction (*P* < 0.01) for PGEM. Calves in LID + MEL had lower PGEM at 4 and 8 h (10.23 and 9.12 pg/mL; 95% CI: 3.01 to 16.34 ng/mL) relative to −24, 0, and 0.5 h (20.38, 27.27, and 22.59 pg/mL, respectively; 95% CI: 14.26 to 33.39 pg/mL; *P* < 0.03). At 4 h, LID + MEL had lower PGEM (10.23 pg/mL; 95% CI: 4.12 to 16.34 pg/mL) relative to ETH (27.08 pg/mL; 95% CI: 19.55 to 34.62 pg/mL; *P* = 0.03). At 8 h, LID + MEL had lower PGEM (9.12 pg/mL; 95% CI: 3.01 to 15.23 pg/mL) relative to ETH and BUP (24.80 and 20.52 pg/mL; 95% CI: 12.68 to 32.33; *P* < 0.03). There was no evidence of a sex effect for PGEM (*P* = 0.27).

Treatment × time interactions were observed for percent change in MNT, VAS score, cortisol, and PGEM concentrations. We chose to investigate the use of a lidocaine cornual block in addition to local infiltration of either lidocaine, ethanol, or bupivacaine liposome suspension based upon the work of Bates et al. (2019), Tapper (2011), and Martin et al. (2022), who investigated the use of the local infiltration method, ethanol as a nerve block for dehorning, and bupivacaine liposome suspension as a nerve block for dehorning, respectively. The investigation of these treatments did not seem to extend the duration of analgesia beyond the currently recommended multimodal approach (AVMA, 2019), including local anesthesia and systemic analgesia such as lidocaine and meloxicam.

For percent change from baseline MNT values, thresholds decreased from baseline (i.e., increased pain sensitivity around the horn buds) beginning at 8 h for BUP, 24 h for ETH, and MEL + LID, and not until 48 h for LID, but there were no significant differences in MNT values among treatments. Calves in all treatments had not returned to baseline values at 72 h, indicating that wounds were likely still sensitive and potentially painful. The VAS scores were significantly higher compared with baseline (i.e., calves exhibited more pain behavior) for ETH, LID, and LID + MEL at 8 and 24 h but only at 8 h for BUP, with VAS declining at 24 h. However, there were no significant differences between treatments.

The LID + MEL treatment resulted in lower cortisol at 2 h relative to BUP. In a recent study, differences in cortisol between administration of a combination of lidocaine and meloxicam and bupivacaine liposome suspension were not observed (Martin et al., 2022); however, bupivacaine liposome suspension was also administered as a cornual block in that study, rather than local infiltration only, ultimately resulting in a larger amount of bupivacaine liposome suspension being administered, which may have had a greater effect on reducing cortisol levels than in the present study. The highest cortisol concentration was observed at 0 and 0.5 h, which is consistent with past research (Martin et al., 2022). The LID + MEL treatment had lower PGEM at 4 and 8 h relative to ETH and ETH and BUP, respectively. A decrease in PGEM in the calves treated with meloxicam was anticipated due to previous findings suggesting that NSAIDs reduce prostaglandin E_2_ over the duration of action of the drug (Stock et al., 2016).

Scoop dehorning followed by cauterization in 5-mo-old calves likely caused more stress and pain relative to calves that are disbudded at a few days old, which likely influenced some of the outcome parameter values in this study relative to previous studies done in younger calves (Adcock and Tucker, 2018; Bates et al., 2019; Martin et al., 2022). The National Dairy FARM Program Animal Care Reference Manual, version 4, requires that calves be disbudded by 8 wk of age (National Milk Producers Federation, 2020). However, male calves with dairy influence destined for beef production may not be raised according to these standards. In 2016, the dairy industry provided approximately 22.7% of US beef; as biotechnology such as sexed semen becomes more prevalently used, the dairy industry's contribution to the US beef industry will remain substantial (DelCurto et al., 2017). Results from the 2017 National Animal Health Monitoring Survey of cow-calf operations showed that only 44% of cow-calf operations dehorned calves before they left the operation, revealing that many beef calves have the potential to be dehorned after weaning (USDA-APHIS-NAHMS, 2020). The need for research into analgesic protocols appropriate for these potentially older dairy and beef animals is apparent, as the pain caused by dehorning may be influenced by age and horn bud width, and effective analgesic protocols for younger animals may not be directly translatable.

Sex effects were observed for MNT and cortisol concentration, with intact males having a higher nociceptive threshold and lower cortisol after dehorning compared with similarly aged heifers. There were more bull calves in the ETH group and fewer in the BUP group relative to the other treatment groups but the sex × treatment interaction was not significant for MNT or cortisol outcomes (*P* > 0.45) when a sex effect was observed. Because there were more heifers than bull calves in the study, sex effects should be interpreted cautiously. Sex differences are not currently well characterized in cattle pain research. Results from human research show that male subjects have higher pain thresholds and tolerance and are less discriminative between painful sensations; in addition, the NSAID ibuprofen has been shown to be less effective in women than in men (Walker and Carmody, 1998; Vallerand and Polomano, 2000). Further investigation into whether these differences exist in cattle among intact males, castrated males, and females is warranted to better quantify and alleviate pain.

At certain time points, LID + MEL reduced cortisol and PGEM more than ETH + LID or BUP + LID administered as a local infiltration and cornual block before scoop dehorning followed by cauterization. The treatments administered did not significantly extend the duration of analgesia beyond the currently recommended multimodal approach, including local anesthesia and systemic analgesia such as lidocaine and meloxicam. Evidence from the current study suggests that sex affects certain pain biomarkers, specifically nociceptive threshold and cortisol concentration, with males having a higher nociceptive threshold and lower cortisol responses.
